# Comparison of miR-106b, miR-191, and miR-30d expression dynamics in milk with regard to its composition in Holstein and Ayrshire cows

**DOI:** 10.5713/ab.23.0427

**Published:** 2024-02-28

**Authors:** Marina V. Pozovnikova, Viktoria B. Leibova, Olga V. Tulinova, Elena A. Romanova, Artem P. Dysin, Natalia V. Dementieva, Anastasiia I. Azovtseva, Sergey E. Sedykh

**Affiliations:** 1 Russian Research Institute of Farm Animal Genetics and Breeding—Branch of the L.K. Ernst Federal Research Center for Animal Husbandry, Pushkin, St. Petersburg, 196625, Russia; 2 Institute of Chemical Biology and Fundamental Medicine, Siberian Branch of the Russian Academy of Sciences, Novosibirsk, 630090, Russia

**Keywords:** Casein, Lactation, Milk Fatty Acids, Reverse Transcription

## Abstract

**Objective:**

Milk composition varies considerably and depends on paratypical, genetic, and epigenetic factors. MiRNAs belong to the class of small non-coding RNAs; they are one of the key tools of epigenetic control because of their ability to regulate gene expression at the post-transcriptional level. We compared the relative expression levels of miR-106b, miR-191, and miR-30d in milk to demonstrate the relationship between the content of these miRNAs with protein and fat components of milk in Holstein and Ayrshire cattle.

**Methods:**

Milk fat, protein, and casein contents were determined in the obtained samples, as well as the content of the main fatty acids (g/100 g milk), including: saturated acids, such as myristic (C14:0), palmitic (C16:0), and stearic (C18:0) acids; monounsaturated acids, including oleic (C18:1) acid; as well as long-, medium- and short-chain, polyunsaturated, and trans fatty acids. Real-time stem-loop one-tube reverse transcription polymerase chain reaction with TaqMan probes was used to measure the miRNA expression levels.

**Results:**

The miRNA expression levels in milk samples were found to be decreased in the first two months in Holstein breed, and in the first four months in Ayrshire breed. Correlation analysis did not reveal any dependence between changes in the expression level of miRNA and milk fat content, but showed a multidirectional relationship with individual milk fatty acids. Positive associations between the expression levels of miR-106b and miR-30d and protein and casein content were found in the Ayrshire breed. Receiver operating characteristic curve analysis showed that miR-106b and miR-30d expression levels can cause changes in fatty acid and protein composition of milk in Ayrshire cows, whereas miR-106b expression level determines the fatty acid composition in Holsteins.

**Conclusion:**

The data obtained in this study showed that miR-106b, miR-191, and miR-30d expression levels in milk samples have peculiarities associated with breed affiliation and the lactation period.

## INTRODUCTION

Cattle domestication has led to the development of various breeds with unique genomic architecture [1], which is phenotypically expressed not only through physical appearance and color [2] variations but also through valuable traits such as milk productivity, and especially milk composition [3]. The ratio of milk components can change because of certain factors, such as diet and season of the year, but these changes differ among breeds [4]. The stage of lactation is another criterion that determines the metabolite composition of milk. Milk is produced by epithelial cells of the mammary gland, which under the influence of both exogenous and endogenous factors undergo numerous physiological changes throughout lactation. Ultimately, these changes determine the intensity of milk component synthesis and, hence, milk composition [5]. Functional genomics studies based on gene network analysis revealed the complexity of molecular adaptation of the mammary gland to lactation. This is assumed to be determined by changes in the transcriptome of mammary epithelial cells during lactogenesis and galactopoiesis [6], whereas the expression level of key candidate genes may vary in animals of different breeds [7]. The emergence of new technologies (DNA microarrays, genome and transcriptome sequencing, etc.) provides a better understanding of mechanisms controlling the regulation of milk production. The use of genomic information has increased selection efficiency in cattle, although the variability of inherited traits is not fully understood [8]. Understanding gene regulation and interactions at the post-transcriptional level can be a useful tool for identifying novel epigenetic markers of productive traits. The complex interaction of genetic and environmental factors determines epigenomic changes, which significantly affect trait expression level [9]. MiRNAs are essential epigenetic components that participate in fundamental processes, including proliferation, embryonic development, tissue differentiation, and apoptosis, and influence lipogenesis, hematopoiesis, and immunity. As a part of the non-coding RNA class, they can regulate up to 60% of gene expression at the post-transcriptional level by binding to complementary RNA molecules, which leads to translation repression or mRNA degradation and, thus, to changes in cellular protein level in different tissue cells [10]. MiRNAs are secreted by body cells and found in all body fluids as part of stable protein or lipid complexes [11]. Being expressed by mammary epithelial cells, they are involved in intracellular communication and signaling pathways at the cellular level, thereby determining the functioning of the mammary gland itself and ultimately the nutrient ratio in milk [12]. Milk as a non-invasive source of miRNAs is an excellent target for studying the mammary transcriptome. Milk fat-derived miRNAs were reported to accurately map the miRNAome of breast tissue [13]. In a recent study [14], the stability of human breast milk miRNAs was experimentally confirmed by treating milk samples with RNase, a low-pH solution, and a triple freeze-thaw cycle (−20°C). Several studies showed that the expression level of miRNA in cow milk depends on housing and environmental conditions, feed ration [15,16], age and breed [17], and the physiological condition of the udder, including mastitis [18]. According to other authors, miRNA expression differs not only in lactating and dry cows but also in groups of cows with high and low milk fat and protein contents [19].

It is well known that Holstein cattle have a high potential for milk productivity, whereas high milk fat content is a distinctive feature of Ayrshire cattle. Modern populations of Ayrshire and Holstein cattle are distinguished by their unique genomic architecture, formed as a result of long-term breeding and artificial selection [20,21]. In this regard, it is relevant to study in detail the contribution of some key milk miRNAs as epigenetic regulators of lactopoiesis and galactopoiesis. MiRNAs including miR-106b (BTA 25; MI0009724), miR-191 (BTA 22; MI0005034), and miR-30d (BTA 14; MI0004747), are presumably involved in the processes regulating the synthesis of protein-fat components of milk, as shown in other studies [22,23]. Overexpression of miR-106b in the mammary gland tissues of Holstein cows resulted in downregulation of the *CDKN1A* gene and alteration of protein synthesis pathways [24]. Although miR-191 is one of the most prevalent miRNAs in bovine mammary gland tissues, its function in lipid and protein synthesis remains unclear. However, its human homologue regulates transcription factors, chromatin remodelers, and cell cycle genes involved in proliferation, apoptosis, differentiation, and migration [25]. Furthermore, miR-191 was identified as a diagnostic marker for breast cancer in women [26]. MiR-30d is a universal miRNA that interacts with many target genes and performs various biological roles. For instance, miR-30d indirectly participates in blood glucose level regulation by activating insulin transcription [27].

It is essential to study the expression patterns of miR-106b, miR-191, and miR-30d in cow milk throughout lactation. To accomplish this aim, we conducted a comprehensive analysis of the aforementioned miRNA expression in the milk of Holstein and Ayrshire cows at different stages of lactation, considering milk composition.

## MATERIALS AND METHODS

### Animal selection

The principles of laboratory animal care were followed, and all procedures were conducted according to the ethical guidelines of the L.K. Ernst Federal Science Center for Animal Husbandry. The protocol was approved by the Commission on the Ethics of Animal Experiments of the L.K. Ernst Federal Science Center for Animal Husbandry (Protocol Number: 2020/2) and the Law of the Russian Federation on Veterinary Medicine No. 4979-1 (14 May 1993).

Two groups of Holstein and Ayrshire cows of 10 animals each were formed for the study, regarding the calving date. Animals were kept on different farms in the same climatic zone under similar conditions. Both groups received balanced mono fodder during the study in accordance with their physiological status.

### Milk sampling

Milk sampling for miRNA extraction was conducted monthly during control milking throughout ten months of the first lactation, specifically in the morning. Milk was sampled in individual tubes without any preservatives; milk samples were immediately cooled (+4°C to +6°C) and transported to the laboratory. Samples were then aliquoted in 5 mL portions, frozen at −80°C and stored until the use. Broad Spectrum Microtabs II preservative was used for analyzing the milk composition. It was added to the samples, stored at +4°C, and delivered to the laboratory within two days from sampling day. Sample analysis was conducted at the center of collective use of scientific equipment of L.K. Ernst Federal Research Center for Animal Husbandry using the CombiFoss 7 infrared analyzer (Foss A/S, Hillerød, Denmark) and included the following parameters: protein (%), fat (%), casein (%), and the main fatty acids (g/100 g of milk). The latter consisted of saturated fatty acids (SFA), including myristic acid (C14:0), palmitic acid (C16:0), stearic acid (C18:0), monounsaturated fatty acids (MUFA), including oleic acid (C18:1), long-chain fatty acids (LCFA), medium-chain fatty acids (MCFA), short-chain fatty acids (SCFA), polyunsaturated fatty acids (PUFA), and trans fatty acids (TFA).

### Extraction and quantification of miRNAs by real-time reverse transcription polymerase chain reaction

MiRNA samples were obtained from 2 mL of milk using the “Total RNA and small RNA isolation kit from “Lira” reagent” (Biolabmix Ltd., Novosibirsk, Russian Federation). RNA concentration and purity (A260/280 ratio) were evaluated using the NanoDrop spectrophotometer (Thermo Fisher Scientific Inc., Waltham, MA, USA). On average, the concentration of isolated RNA was 50 to 180 ng/μL. MiRNA expression levels were measured using real-time stem-loop one-tube real-time reverse transcription polymerase chain reaction (RT-qPCR), proposed and described previously by Varkonyi-Gasic et al [28]. For reverse transcription (RT), we used stem-loop primers and a TaqMan probe, similar to UPL-21 (F. Hoffmann-La Roche Ltd., Basel, Switzerland), which was developed as part of this study. The RT reaction was performed using the “Reverse transcriptase M-MuLV-RH” kit (Biolabmix Ltd., Russian Federation) in a volume of 10 μL. Then, 2 μL of the obtained miRNA solution was used as a matrix in the following regime: 30 min 16°C; 45 cycles of 30 s 30°C; 30 s 42°C; 1 s 50°C; and the final step of 5 min 85°C. The resulting cDNA-containing solution was used as a matrix for real-time qPCR. The reaction was performed in a volume of 20 μL, and the amplification of PCR products was analyzed using QuantStudio 5 Real Time PCR System (Thermo Fisher Scientific Inc., USA). The PCR mix consisted of 2× BioMaster HS-qPCR reaction mix (Biolabmix Ltd., Russian Federation), 0.2 μmol of forward and reverse primers, 0.1 μmol TaqMan probe and 2 μL of RT-PCR products. Primers were designed using “miRNA Primer Design Tool” program (https://genomics.dote.hu:8080/ mirnadesigntool/processor) and synthesized at ICBFM SB RAS (Novosibirsk, Russian Federation). The amplification protocol was as follows: 10 min 95°C; 40 cycles of 15 s 95°C; 25 s 60°C. The amplification quality was evaluated by the amplification curve distribution depending on the initial matrix concentration. For each sample RT-qPCR reactions were performed in 3 repeats. A series of dilutions from 10^−1^ to 10^−8^ ng/μL of synthetic miRNA bta-miR-191 (IHBFM SB RAS, Novosibirsk, Russian Federation) were used as reference samples for calibration plotting.

The nucleotide sequences of the primers and TaqMan probe are presented in Table 1.

### Statistical analysis

The obtained results were processed using Statistica.10 (StatSoft, Inc., Tulsa, OK, USA) and GraphPad Prism 12.0 (GraphPad Software Inc., La Jolla, CA, USA) application packages. Quantitative data were tested for normality using the Kolmogorov-Smirnov criterion. The statistical significance of the parameter difference between groups was assessed using nonparametric analysis methods, specifically the Kruskal-Wallis H-criterion (H-test) when comparing several groups, and the Mann-Whitney criterion when comparing two groups. Differences were considered statistically significant if p≤0.05 (after adjusting for the number of comparisons). The data were then analyzed using two-way analysis of variance (ANOVA) to test the main effects of the factors “Farm” (combination of breed, housing system, and feed ration factors) and “month of lactation” on milk composition (Table 2).

“Plot Tukey” plots were constructed to visualize the relative expression level of microRNAs, using GraphPad Prism 12.0 software. The logarithm function for the y axis (log^10^) was applied to improve the distribution of trait values. Spearman’s coefficient was used for correlation analysis and construction of the heatmap in GraphPad Prism 12.0 software. The critical significance level was set to p<0.05. Receiver operating characteristic (ROC) curves of miRNA data were analyzed using GraphPad Prism 12. In all cases, the critical significance level was also set to p<0.05.

Principal components and classification analysis (PCC) based on the covariance matrix was performed on normalized data using Statistica.10 software (StatSoft Inc., USA). Changes in the expression of individual miRNAs were calculated using the 2dCt (delta Cycle threshold) method [29]. Target genes were searched and “Network” (multi-association network integration) plotting was performed in miRWalk database [30] (https://mirwalk.umm.uni-heidelberg.de/). Annotation of signaling pathways for the identified target genes was performed using the Kyoto encyclopedia of genes and genomes (KEGG) genomic browser (https://www.genome.jp/kegg/). Enriched signaling pathways for the identified target genes were annotated and visualization of the results was performed using ShinyGo 0.77 (https://bioinformatics.sdstate.edu/go/).

## RESULTS

### Phenotype analysis

**Figure 1:**
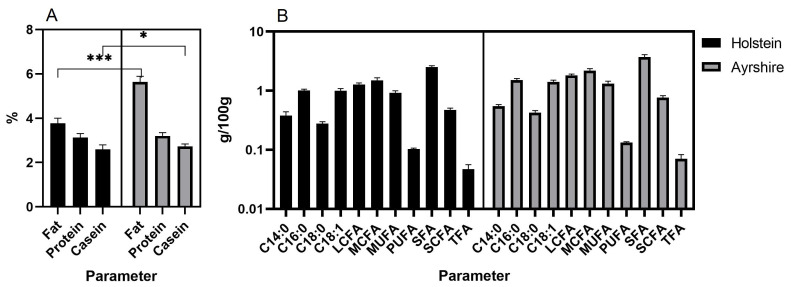
Milk characteristics of the studied cow groups by total values for ten months of lactation (Median; with 95% CI). (A) Fat, protein, casein; *** p<0.001; * p<0.05. (B) C14:0, C16:0, C18:0, C18:1, LCFA, MCFA, MUFA, PUFA, SFA, SCFA, TFA (p<0.05 for all constituents). LCFA, long-chain fatty acid; MCFA, medium-chain fatty acids; MUFA, monounsaturated fatty acids; PUFA, polyunsaturated fatty acids; SFA, saturated fatty acids; SCFA, short-chain fatty acids; TFA, trans fatty acids.

As a result, milk composition data for ten months of lactation were obtained in both groups of animals (Figure 1). Milk of Ayrshire cows was found to be higher in fat (p<0.001), casein (p<0.05) (Figure 1A) and all fatty acids (p<0.05) (Figure 1B) compared to Holsteins.

**Figure 2:**
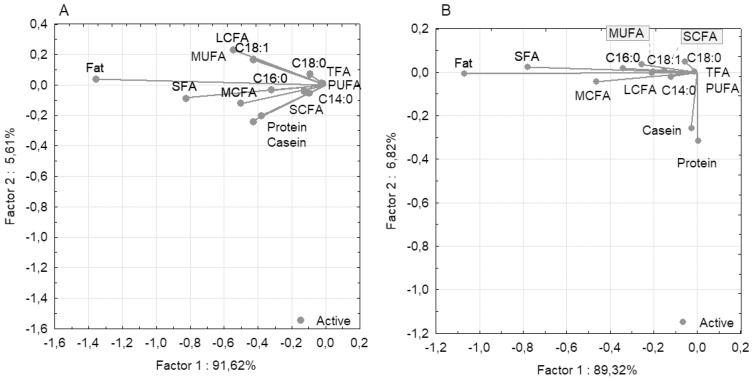
Graphical visualization of covariance between milk components based on principal components and classification analysis (PCC): (A) Holstein breed; (B) Ayrshire breed. LCFA, long-chain fatty acid; MCFA, medium-chain fatty acids; MUFA, monounsaturated fatty acids; PUFA, polyunsaturated fatty acids; SFA, saturated fatty acids; SCFA, short-chain fatty acids; TFA, trans fatty acids.

PCC analysis for milk traits demonstrated that the first component explained 91.62% of the total phenotypic variability for all 14 traits for the Holstein breed and 89.32% for the Ayrshire breed (Figure 2). For both breeds, the fat trait was isolated and demonstrated high negative loading for the first component (−1.359 for Holstein and −1.070 for Ayrshire). SCFA and TFA had low negative loadings (−0.130 and −0.023 for Holstein, and −0.151 and −0.013 for Ayrshire, respectively). Notably, the protein and casein traits diverged in the Ayrshire group, with a slightly positive loading for protein (0.003) and a slightly negative loading for casein (−0.027). For the second component, both traits demonstrated high negative loadings (protein −0.317 and casein −0.257), indicating their divergence relative to the fatty acid components.

**Figure 3:**
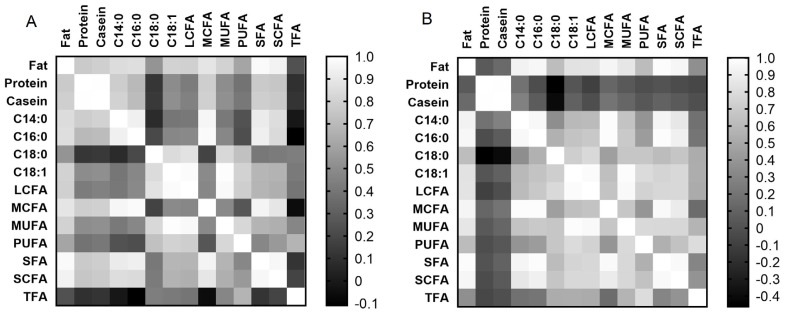
Correlation matrix for analyzed milk traits in cows: (A) Holstein breed; (B) Ayrshire breed.

Correlation matrix analysis showed (Figure 3A, 3B) that both breeds had similar correlations between milk fat content and fatty acid composition, as well as between individual fatty acids (at least p<0.05). However, only Holstein cows showed a positive correlation of protein and casein contents with fat content (r = 0.768 and r = 0.789, respectively, at p< 0.001) and fatty acids in milk (at least p<0.05), compared to Ayrshire cows. For the Ayrshire breed, a negative correlation was found between milk protein and casein with C18:0 (r = −0.462 and r = −0.409 respectively, p<0.05) and a positive correlation between casein and C14:0 (r = 0.274, p<0.05). Thus, correlation analysis showed significant differences in the interdependency of milk protein and casein with the content of fat and fatty acids in both breeds.

Table 3 demonstrates the variation in milk constituent values during lactation for the Holstein and Ayrshire breeds. In Holstein cattle, all milk constituents exhibited high variability, except PUFA (p = 0.0515). Milk composition in the Ayrshire group was relatively stable during the observation period. Significant variability was found only for constituents such as protein, casein, C14:0, C18:0, and TFA (at least p<0.05).

### Analysis of miR-106b, miR-191, and miR-30d expression levels

**Figure 4:**
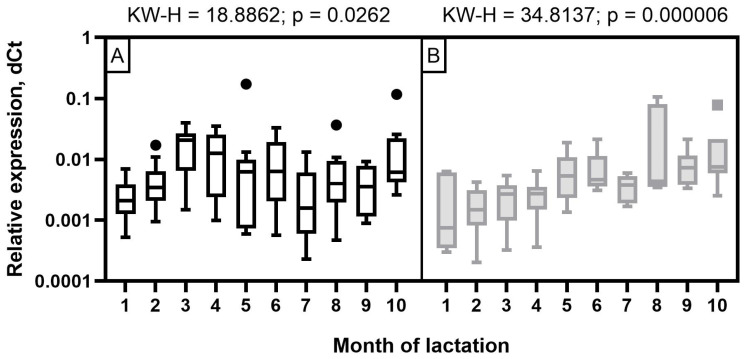
Dynamics of miR-106b relative expression level in milk samples of (A) Holstein and (B) Ayrshire cows during lactation. KW-H, Kruskal-Wallis H-test.

The expression level of miR-106b in milk samples of Holstein cows showed a wave-like pattern of variation throughout the observation period (Figure 4). A low expression level was detected at the beginning of the first two months of lactation. It increased significantly by the third and fourth months (p< 0.05 to 0.001) and decreased again by the seventh and ninth months with a subsequent increase by the end of lactation (p<0.05 to 0.001). In the milk of Ayrshire cows, miR-106b had a low expression level in the first four months with an increase from five to seven months and reaching maximum values in the late period of lactation (p<0.05 to 0.001).

**Figure 5:**
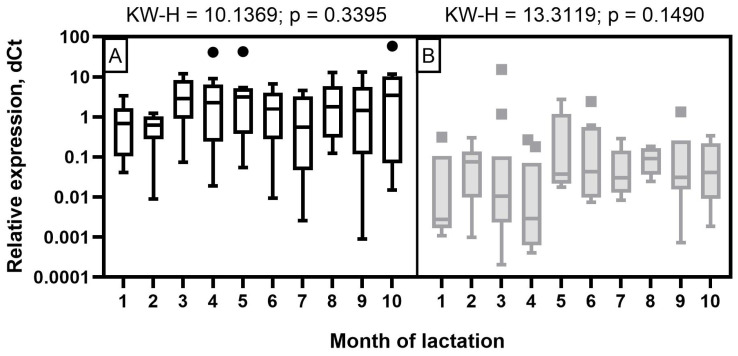
Dynamics of miR-191 relative expression level in milk samples of (A) Holstein and (B) Ayrshire cows during lactation. KW-H, Kruskal-Wallis H-test.

For miR-191 in Holstein cows, a statistically significant increase in expression was found only by the third month of lactation, as well as a tendency to higher expression values at the fourth and fifth month, compared to the second month. In the milk of Ayrshire cows, statistically significant increases in miR-191 were found at the fifth and sixth months of lactation, compared to first month (p<0.05 to 0.001) and at 5 to 7 months compared to fourth month (p<0.05 to 0.001) (Figure 5). In general, the obtained values of miR-191 relative expression level were significantly lower for Ayrshire cows, than for Holstein cows.

**Figure 6:**
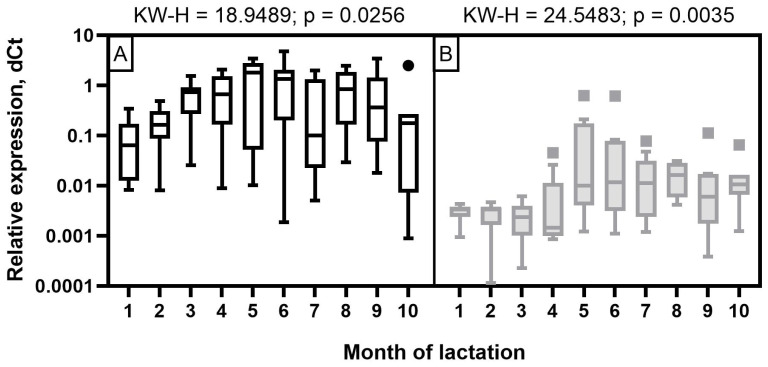
Dynamics of miR-30d relative expression levels in milk samples from (A) Holstein and (B) Ayrshire cows during lactation. KW-H, Kruskal-Wallis H-test.

The expression level of miR-30d in Holstein cows was low at the beginning of lactation, with its subsequent increase starting from three months and reaching its maximum by 5 to 6 months, which corresponds to mid-lactation (p<0.05 to 0.001). As for Ayrshire cows, miR-30d expression was characterized by low values in the first four months of lactation, a significant increase at 5 to 6 months with a smooth subsequent decrease, but not reaching the level of 1 to 4 months (p<0.05 to 0.001) (Figure 6). With certain dynamics of miR-30d expression level during the observation period, the values were lower in milk samples from Ayrshire cows compared to Holsteins.

### Correlation analysis

Correlation analysis of the Holstein breed data showed a positive correlation between the expression levels of miRNAs (miR-106b – miR-191, r = 0.799; miR-106b – miR-30d, r = 0.752; miR-30d – miR-191, r = 0.841; all at p<0.05). Nevertheless, associative relationships of analyzed miRNAs with milk fat components had their own peculiarities (Table 4). A statistically significant negative correlation was found between miR-106b expression level and the content of C18:1 (p<0.05), SFA (p<0.05), SCFA (p<0.05), and showed a similar tendency with fat, C18:0 (p = 0.059), LCFA (p = 0.055), and MCFA (p = 0.071). The expression level of miR-191 was negatively correlated with C18:0 (p<0.01), C18:1 (p<0.05), and LCFA (p<0.05). A negative correlation was also found for miR-30d expression level with C18:0 content (p<0.01), and, with a tendency towards reliability with C18:1 (p = 0.063) and LCFA (p = 0.070) (Table 4).

In Ayrshire cattle, correlation analysis revealed a positive correlation between miR-106b expression level and protein (p<0.001), casein (p<0.001), and a negative correlation with C18:0 (p<0.01). Similar results were obtained for miR-30d, whose expression level was positively correlated with protein (p<0.001) and casein (p<0.001) and negatively correlated with C18:0 (p<0.05). A multidirectional relationship with TFA level (p<0.01) was shown for miR-191 (Table 4). Similar to Holstein cattle, these miRNAs had a unidirectional positive correlation among themselves (miR-106b – miR-191, r = 0.446; miR-106b – miR-30d, r = 0.369; miR-30d – miR-191, r = 0.493; all at p<0.05).

**Figure 7:**
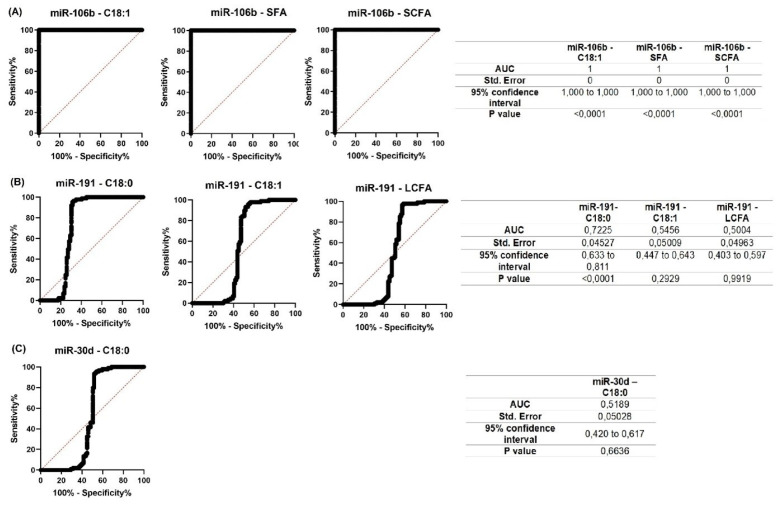
Receiver operating characteristic (ROC) curves obtained from the most efficient microRNA predictor for several milk constituents of Holstein cows. (A) for miR-106b; (B) for miR-191; (C) for miR-30d. AUC, area under the curve.

**Figure 8:**
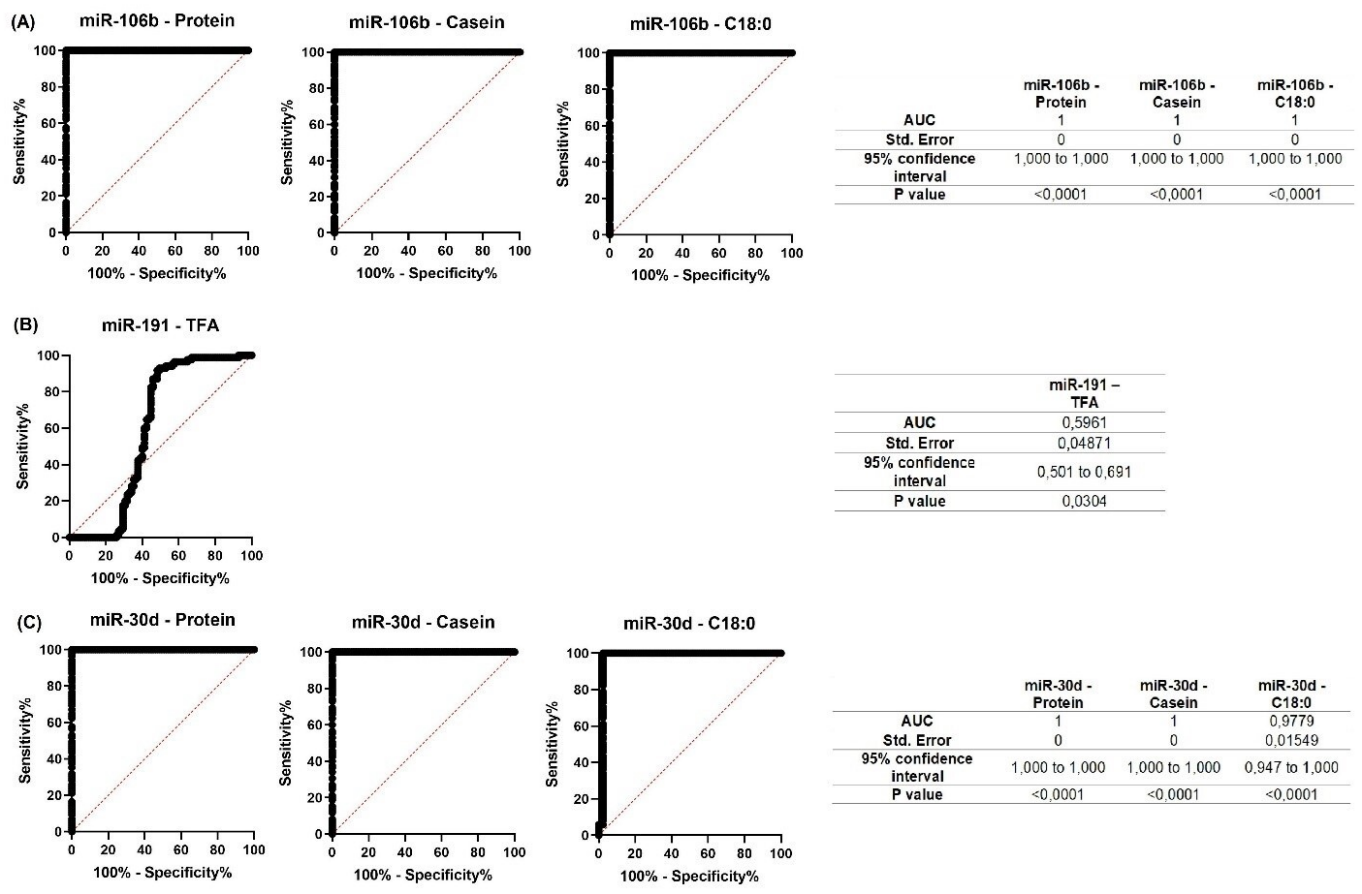
Receiver operating characteristic (ROC) curves obtained from the most efficient microRNA predictor for several milk constituents of Ayrshire cows. (A) for miR-106b; (B) for miR-191; (C) for miR-30d. AUC, area under the curve.

For all miRNAs that showed significant correlations with milk constituents, ROC curves were created (Figures 7; 8). The purpose of analyzing the ROC curves was to determine the extent to which miRNA expression levels may have a prognostic effect for the milk constituents. We found that for Holstein cows’ milk, miR-106b had high predictive values (AUC = 1) for C18:1, SFA and SCFA content; miR-191 had moderate to low predictive value (0.5<AUC<0.7) for C18:0, C18:1, and LCFA content, whereas miR-30d had low predictive value for C18:0 (0.5<AUC<0.6) (Figure 7). For Ayrshire cows’ milk, miR-106b and miR-30d had high predictive values (AUC = 1) for protein, casein and C18:0 content, while miR-191 showed low values (0.5<AUC<0.6) for TFA content (Figure 8).

**Figure 9:**
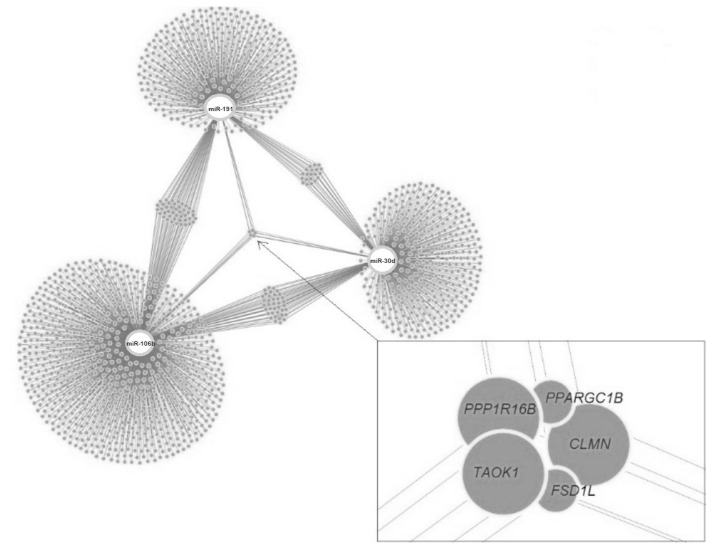
MiRNAs and all their target genes, including “central module” genes.

**Figure 10:**
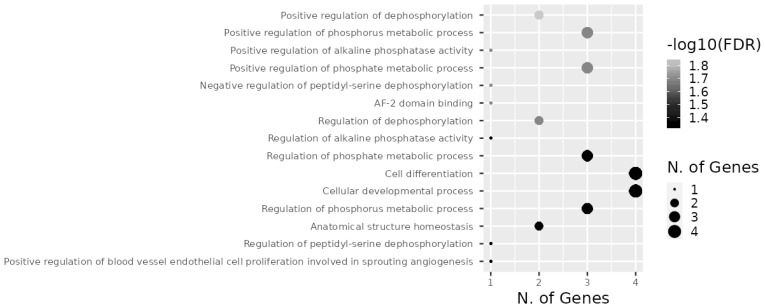
Enriched signaling pathways for miR-106b, miR-191 miR-30d “central module” target genes.

Target genes of the investigated miRNAs were searched for in the miRWalk database (Figure 9). A total of 1,531 genes were identified, 5 of which were localized in the central module and were common target genes for all three miRNAs (*TAOK1*, *FSD1L*, *PPARGC1B*, *PPP1R16B*, *CLMN*). A total of 15 signaling pathways were predicted for *TAOK1*, *PPARGC1B*, *PPP1R16B*, and *CLMN* genes (Figure 10) with the exception of the *FSD1L* gene. The biological significance of miRNAs was assessed by annotating the most important reactome pathways in the miRWalk database (Table 5). A total of 97 pathways was identified for miR-106b, 41 pathways for miR-191, and 61 pathways for miR-30d. For all three miRNAs, involvement in the pathways of lipid and protein metabolism was observed, whereas only miR-106b was involved in the pathways of fatty acid metabolism, and amino acids and their derivatives metabolism.

## DISCUSSION

Effective breeding of dairy cattle requires considering the peculiarities of milk composition in different breeds. Milk composition, according to Schwendel et al [31], depends on a variety of paratypical and genetic factors, so the determination of paratypical factors is of great practical importance for industrial production. In addition, breed and housing system (including feed ration) constitute the minimum set of factors to be considered when comparing milk samples. Therefore, the statistical analysis takes into account a complex factor that includes both breed and housing system. Breed can be a decisive factor that largely accounts for differences between housing systems [32]. Although we obtained differences between cow groups in protein and casein content, the “farm” factor did not significantly affect the content of these components in milk. This result, apparently, can be explained by the fact that both farms provided conditions sufficient for the same manifestation of the studied traits, as well as by the fact that cows were at the same stage of lactation and received a balanced mono fodder in accordance with their physiological status. As our data show (Table 3), the content of protein-fat constituents of Holstein cows’ milk changed significantly during 10 months of lactation, and only the level of PUFA and TFA remained relatively stable. In Ayrshire cows, only the values of milk protein, casein, C14:0, C18:0, and TFA content significantly changed (at least at p<0.05). In general, both breeds showed an increase in the content of all investigated milk constituents until late lactation (month 7 to 8), which is probably due to physiological changes caused by late lactation and decreased milk yield. ANOVA analysis revealed that the factor “month of lactation” had no significant effect on C18:1, LCFA, PUFA, and SCFA content in milk of both breeds during the first lactation (Table 2). The combined effect of the two factors “farm+month of lactation” was significant for all analyzed milk constituents in both breeds.

The milk composition characteristics of Holstein and Ayrshire cattle may be defined by differences in their genomic architecture [21]. This factor determines the presence of different sets of candidate genes, which are phenotypically expressed through milk traits [33]. Higher fat, protein and casein content in milk, compared to Holstein cows, is a specific breed trait of Ayrshire cattle (p<0.01 to 0.001) [34]. Similar interbreed differences were observed in our study, where milk from Ayrshire cows contained on average 1.82% more fat (p<0.001), 0.06% more protein and 0.08% more casein (p<0.05). Significant differences in milk fat content appear to cause differences in fatty acid content as well (p<0.001). PCC analysis for both breeds showed clustering of fatty acids with high correlation coefficients (p<0.05 to 0.001). For the Ayrshire breed, PCC analysis demonstrated clustering of protein and casein traits relative to fat and fatty acids, with no significant correlations between these traits. For Holstein cows, a unidirectional correlation between protein, casein, and fat components of milk was established. The identified differences in the ratio of protein-fat components in these breeds presumably determine the technological properties of milk [34].

The results obtained in this research, based on the real-time stem-loop RT-qPCR data, revealed different expression patterns of miR-106b, miR-191, and miR-30d in milk samples during lactation in Ayrshire and Holstein cows. Against the background of considerable fluctuations in protein-fat components (p<0.05 to 0.001), except for PUFA, the expression levels of miR-106b, miR-191, and miR-30d in milk samples of Holstein cows were reduced in the first two months, whereas in Ayrshire cows they were reduced by the fourth month. In contrast, the milk composition of the latter was relatively stable throughout lactation, except for protein, casein, C14:0, C18:0, and TFA (at least p<0.05).

Correlation analysis demonstrated that both breeds exhibited positive relationships between the expression levels of miR-106b, miR-191, and miR-30d with each other, while displaying negative correlations with several fatty acids, which is consistent with the generally accepted statement that miRNA is a negative post-translational regulator. Moreover, among the components characterizing milk fat composition, only C18:1, C18:0, and LCFA were negatively correlated with each of the three miRNAs in milk from Holstein cows. The Ayrshire breed also exhibited negative correlations between milk fat components and miRNAs, including miR-106b and miR-30d with C18:0, and miR-191 with TFA, which was absent in milk samples from Holstein cows. The Ayrshire breed specificity was the positive correlation of miR-106b and miR-30d with milk protein components. According to the literature, miRNAs do not always act as negative post-translational regulators. Previously, while studying the functional role of 11 miRNAs in the processes of milk fat synthesis regulation in epithelial cells of the goat mammary gland, it was found that increased expression of three miRNAs (miR-23a, miR-103, and miR-200a) led to activation of transcription and translation of their target genes [35].

The study of miRNAs is important not only for understanding galactopoiesis, but also for human health. Fatty acids (FA) associated with the expression of the studied miRNAs are important for healthy human diet. Stearic acid (C18:0) exhibits a neutral or beneficial effect on blood cholesterol levels. It can also be desaturated to oleic acid that, in turn, is associated with lower risk of cardiovascular disease, diabetes, and obesity, as it can improve the lipid profile, insulin sensitivity, and inflammatory markers [36].

SCFA can influence the gut-brain axis, which is the bidirectional communication between the gastrointestinal tract and the central nervous system, by affecting the production and release of neurotransmitters, hormones, and cytokines. SCFA can also modulate the energy balance, glucose homeostasis, lipid metabolism, and immune function by activating specific receptors, such as G-protein coupled receptors (*GPR41*, *GPR43*, and *GPR109A*) and free fatty acid receptors (*FFAR2* and *FFAR3*), or by inhibiting histone deacetylases [37].

TFA have been associated with increased risk of cardiovascular disease, diabetes, obesity, and cancer, as they can adversely affect the lipid profile, insulin resistance, inflammation, and oxidative stress [38]. However, the effects of TFA may vary depending on their type and dose. Industrial TFA (iTFA), on the other hand, have been shown to have more detrimental effects on health, compared to ruminant TFA (rTFA), as they can increase the levels of LDL cholesterol, triglycerides, and lipoprotein(a), decrease the levels of HDL cholesterol, and impair the endothelial function [39].

SFA can also influence the gut-brain axis, energy balance, glucose homeostasis, lipid metabolism, and the immune function, depending on their chain length and concentration [40].

Cow’s milk protein is a complex mixture of bioactive peptides and proteins, which can modulate various physiological systems in humans, such as the immune, cardiovascular, gastrointestinal, and nervous systems [41]. Casein, which constitutes about 80% of the total protein content of cow’s milk, influences the physical properties of milk and dairy products, such as viscosity, stability, and texture, which are relevant for processing and storage of dairy products [42].

Therefore, predicting the component composition of cow’s milk is crucial for enhancing the quality of human nutrition. ROC curves data suggest that miRNAs can be used as a prognostic marker in the early prediction of cow’s milk composition. Thus, in our study, miR-106b had high predictive values for C18:1, SFA, and SCFA (AUC = 1) in Holstein cows, and for protein, casein, and C18:0 (AUC = 1) in Ayrshire cows. In contrast, miR-30d had high predictive values (AUC = 1) for protein, casein and C18:0 only in Ayrshire cows. In the presence of some reliable correlations in both cow groups, miR-191 showed low predictive value, and therefore cannot be recommended as a predictive marker of protein-fat composition of milk.

The miRNAs’ biological features, specifically their ability to target dozens of genes, suggest their involvement in many signaling pathways. Understanding the mechanisms of genetic and epigenetic regulation of processes affecting the synthesis of milk components may improve milk productivity in cows. More than 6,000 genes regulate milk synthesis processes, and expression of these genes is observed in both mammary gland tissues and other types of tissues [43].

In our study, 1,531 target genes involved in nearly 200 reactome pathways were identified for miR-106b, miR-191, and miR-30d (Table 5). The target gene network plot identified a cluster of 5 genes that were targets for all miRNAs analyzed in the study, i.e. *TAOK1*, *FSD1L*, *PPARGC1B*, *PPP1R16B*, and *CLMN*.

The *TAOK1* gene, serine/threonine-protein kinase TAO1, is a MAP3K protein kinase that, through regulation of the mitogen-activated protein kinase pathway, modulates a significant number of cellular processes. *TAOK1* was previously identified as one of the functional candidate genes for immunoglobulin G (IgG) and IgM immunoglobulin content in the colostrum and serum of Holstein cows [44], confirming its involvement in the synthesis of milk components.

The *FSD1L* gene, fibronectin type III and SPRY domain-containing protein 1, encodes type 2 cystatins, members of the cystatin family of intracellular and extracellular protease inhibitors. The function of this gene is poorly understood; however, in a study by Tahir et al [45], a single nucleotide polymorphism (SNP) located near the genomic region comprising the *FSD1L* gene (BTA8) was associated with heifer fertility traits, which are known to be negatively correlated with cow milk production traits.

The *PPARGC1B* gene, peroxisome proliferator-activated receptor gamma coactivator 1-beta isoform X2, is known to be involved in the Insulin resistance biological pathway (bta04931), as well as to regulate glucose homeostasis and mitochondrial biogenesis. Its participation in the regulation of milk fat synthesis was previously confirmed [46]. Earlier in a GWAS study, the *PPARGC1B* gene was identified as significant for milk production traits in Sahiwal-Tharparkar cows [47]. In another study, addition of high oleic sunflower seeds to the cows’ ration was associated with an increase in the proportion of C18:0 and C18:1 in milk and an increase in *PPARGC1B* transcriptional activity in the mammary gland [48].

The *PPP1R16B* gene, protein phosphatase 1 regulatory inhibitor subunit 16B, is involved in biological processes such as endothelial barrier establishment and positive regulation of blood vessel endothelial cell proliferation involved in sprouting angiogenesis. According to Pszczola et al [49], this gene was associated with cows’ methane production level, which, according to the authors, is caused by the presumed impact of this gene on digestion and nutrient assimilation processes. At the same time, when taking phenotypic traits into account, the researchers also made adjustments to the fat and protein content of milk. Earlier studies have linked fatty acid levels in milk to methane production in cows; in particular C18:1 level had a negative correlation with CH_4_ [50].

The *CLMN* gene, calmin isoform X1, has been identified as one of the genes sensitive to the vitamin A metabolite all-trans retinoic acid (atRA), which regulates the development of the nervous system during embryonic development [51]. Although the role of *CLMN* gene in regulating the synthesis of protein-fat constituents of milk is not clear, there is evidence supporting its involvement in lipid metabolism. GWAS analysis revealed an association of SNP in the *CLMN* gene with changes in total cholesterol and plasma LDL-cholesterol levels in patients taking statins [52]. In cattle studies, the *CLMN* gene haplotype was associated with meat marbling score in Korean beef cattle Hanwoo [53].

Some target genes detected by reactome pathways analysis (Table 5) were associated with certain milk constituents. Polymorphic variants of *GPAM* gene were associated with milk fat, protein and dry matter content in Holstein cows’ milk [54]. A method combining GWAS with a gene-centric approach revealed that the *PYCR1* gene is included in genomic regions associated with fatty acid profile and milk fat content in cow milk [55].

Our data provide new insights into the relationship between miRNA expression and milk composition and suggest that miR-106b, miR-191, and miR-30d play a potentially significant role in galactopoiesis in both Holstein and Ayrshire cattle. The differences observed in the expression patterns of the analyzed miRNAs may be caused by specific genomic architectures of the studied breeds. This is probably caused by the fact that in a number of candidate genes the phenotypic effect varies both by lactation number and dairy cattle breed.

Since different breeds may have different genetic and epigenetic backgrounds that affect miRNA expression and milk composition, it would be advisable to compare the results with other dairy cattle breeds (Jersey, Danish Red) and combined productivity breeds (Brown Swiss, Simmental). Identification of the target genes and pathways of these miRNAs in mammary tissue and milk would also be useful, as well as confirmation of their action by functional analyses such as knockdown or overexpression experiments. This will help to identify the molecular mechanisms of lactation, as well as the epigenetic mechanisms controlling milk composition and quality in the studied breeds, which may benefit the dairy industry and consumers.

Analysis of miRNA expression levels can also be used to predict milk composition and quality or to select cows with desired milk characteristics. In addition, external factors like diet and environment, can alter miRNA expression levels to enhance milk composition and quality. These applications will require further validation and optimization in future studies.

## CONCLUSION

Our results showed that milk from Holstein and Ayrshire breeds had different protein-fat component ratios. The expression levels of miR-106b, miR-191, and miR-30d in milk samples differed depending on the lactation period and breed. The correlation analysis did not reveal a correlation between changes in miRNA expression level and milk fat content. However, a negative relationship with several milk fatty acids in both breeds was observed. A positive correlation between the expression levels of miR-106b and miR-30d and the total protein and casein content was found in the milk of Ayrshire cows. This finding suggests that different epigenetic mechanisms regulating the synthesis of milk components in mammary gland tissues might exist in different breeds. The calculated AUC values in ROC curves analysis showed that changes in the expression of miR-106b and miR-30d can be a prognostic marker for the milk composition in terms of C18:0, protein and casein content in Ayrshire cows. In Holstein cows, the expression level of miR-106b can also be a prognostic factor, but for fatty acid composition of milk (C18:1, SFA, and SCFA).

The analysis of the relative expression levels of key miRNAs in dairy cow milk contributes to a better understanding of epigenetic factors affecting milk composition and expands our knowledge of lactogenesis and galactopoiesis. The proposed real-time RT-qPCR method using stem-loop primers and the designed TaqMan probe is a universal tool for milk miRNA analysis, which other researchers can use for further characterization of these miRNAs.

## Figures and Tables

**Table 1:** Nucleotide sequence of the primers and TaqMan probe used for real-time reverse transcription polymerase chain reaction

Oligo name	Primer sequence
mimetic miR-191	CAACGGAAUCCCAAAAGCAGCUG
Universal reverse primer	GTGCAGGGTCCGAGGT
TaqMan probe	[FAM]TGGCTCTGGTGCGAATAC[BHQ1]
miR-191 forward primer	CAACGGAAUCCCAAAAGCAGCUG
miR-191 Stem-loop primer	GTTGGCTCTGGTGCAGGGTCCGAGGTATTCGCACCAGAGCCAACCAGCTG
miR-106b forward primer	UAAAGUGCUGACAGUGCAGAU
miR-106b Stem-loop primer	GTTGGCTCTGGTGCAGGGTCCGAGGTATTCGCACCAGAGCCAACATCTGC
miR-30d forward primer	UGUAAACAUCCCCGACUGGAAGCU
miR-30d Stem-loop primer	GTTGGCTCTGGTGCAGGGTCCGAGGTATTCGCACCAGAGCCAAC AGCTTC

**Table 2:** The effect of paratypic factors on phenotypic variability of cow’s milk composition

Constituents	Factors								

	Farm			Month of lactation			Farm+month of lactation		
		
	R^2^	F	p-value	R^2^	F	p-value	R^2^	F	p-value
Fat	0.352	93.534	0	0.133	2,785	0.005	0.572	10.853	0
Protein	0.005	0.908	0.342	0.441	14,349	0	0.506	8.301	0
Casein	0.012	2.097	0.149	0.453	15,074	0	0.533	9.233	0
C14:0	0.301	73.979	0	0.262	6,482	0	0.642	14.522	0
C16:0	0.344	90.273	0	0.156	3,364	0.001	0.562	10.407	0
C18:0	0.311	77.713	0	0.115	2,357	0.016	0.537	9.39	0
C18:1	0.186	39.402	0	0.082	1,622	0.113	0.37	4.755	0
LCFA	0.204	44.169	0	0.086	1,714	0.089	0.403	5.463	0
MCFA	0.338	87.969	0	0.19	4,277	0	0.606	12.441	0
MUFA	0.168	34.783	0	0.096	1,94	0.05	0.352	4.394	0
PUFA	0.203	43.87	0	0.033	0.622	0.777	0.347	4.298	0
SFA	0.394	111.69	0	0.127	2,659	0.007	0.611	12.716	0
SCFA	0.45	140.978	0	0.075	1,475	0.161	0.638	14.305	0
TFA	0.075	14.012	0	0.109	2,224	0.023	0.253	2.746	0

R^2^, coefficient of determination; F, Fisher’s criterion; p, significance level.

The factor “farm” had no significant effect on the content of protein and casein in milk, the factor “month of lactation” - on the content of C18:1, LCFA, PUFA, and SCFA. At the same time, the combined effect of the factors “farm+month of lactation” had a significant effect on all analyzed milk parameters.

LCFA, long-chain fatty acid; MCFA, medium-chain fatty acids; MUFA, monounsaturated fatty acids; PUFA, polyunsaturated fatty acids; SFA, saturated fatty acids; SCFA, short-chain fatty acids; TFA, trans fatty acids.

**Table 3:** Dynamics of the main milk constituents of Holstein and Ayrshire cattle during lactation

Constituents		Lactation month										p-value

		1	2	3	4	5	6	7	8	9	10	
Holstein breed												
Fat (%)	Mean	3.11	2.86	2.60	3.64	3.84	4.94	4.36	4.14	4.42	4.57	<0.001
	SD	0.34	0.41	0.49	0.69	0.81	1.95	0.59	0.71	0.46	0.59	
Protein (%)	Mean	2.84	2.49	2.73	2.97	3.27	3.31	3.44	3.46	3.56	3.45	<0.001
	SD	0.09	0.21	0.17	0.20	0.29	0.38	0.29	0.31	0.27	0.33	
Casein (%)	Mean	2.35	2.06	2.28	2.50	2.75	2.79	2.90	2.91	3.01	2.91	<0.001
	SD	0.09	0.18	0.13	0.16	0.25	0.31	0.24	0.26	0.21	0.27	
C14:0 (g/100 g)	Mean	0.26	0.25	0.32	0.36	0.40	0.51	0.49	0.47	0.50	0.48	<0.001
	SD	0.06	0.04	0.03	0.07	0.07	0.09	0.06	0.09	0.05	0.05	
C16:0 (g/100 g)	Mean	0.76	0.84	0.81	0.89	0.96	1.36	1.19	1.09	1.17	1.20	<0.001
	SD	0.10	0.10	0.11	0.20	0.16	0.41	0.14	0.21	0.12	0.13	
C18:0 (g/100 g)	Mean	0.34	0.31	0.18	0.30	0.27	0.36	0.25	0.26	0.29	0.34	<0.05
	SD	0.12	0.08	0.07	0.06	0.06	0.20	0.04	0.04	0.04	0.05	
C18:1 (g/100 g)	Mean	1.02	0.93	0.66	1.02	1.04	1.30	1.06	1.03	1.09	1.24	<0.05
	SD	0.30	0.22	0.17	0.19	0.22	0.78	0.17	0.17	0.10	0.21	
LCFA (g/100 g)	Mean	1.24	1.19	0.77	1.33	1.28	1.66	1.32	1.28	1.37	1.60	<0.001
	SD	0.42	0.30	0.27	0.28	0.31	0.97	0.21	0.20	0.13	0.21	
MCFA (g/100 g)	Mean	1.15	1.10	1.12	1.33	1.48	2.05	1.86	1.74	1.86	1.84	<0.001
	SD	0.18	0.14	0.17	0.30	0.27	0.58	0.22	0.36	0.20	0.21	
MUFA (g/100 g)	Mean	0.92	0.85	0.65	0.97	0.98	1.23	0.97	0.96	1.01	1.17	<0.05
	SD	0.29	0.20	0.18	0.17	0.21	0.76	0.15	0.15	0.08	0.20	
PUFA (g/100 g)	Mean	0.10	0.10	0.08	0.11	0.11	0.11	0.10	0.11	0.11	0.12	0.0512
	SD	0.02	0.01	0.02	0.01	0.02	0.06	0.02	0.01	0.02	0.03	
SFA (g/100 g)	Mean	2.03	1.89	1.77	2.32	2.51	3.30	2.99	2.80	3.02	2.99	<0.001
	SD	0.19	0.25	0.31	0.49	0.53	1.04	0.41	0.53	0.34	0.35	
SCFA (g/100 g)	Mean	0.43	0.36	0.33	0.46	0.51	0.61	0.60	0.56	0.61	0.57	<0.001
	SD	0.05	0.06	0.07	0.11	0.13	0.17	0.10	0.11	0.10	0.11	
TFA (g/100 g)	Mean	0.06	0.04	0.05	0.06	0.07	0.05	0.04	0.05	0.05	0.06	<0.05
	SD	0.02	0.02	0.03	0.02	0.02	0.06	0.03	0.02	0.02	0.02	
Ayrshire breed												
Fat (%)	Mean	5.24	5.22	6.06	6.20	6.02	6.36	5.32	5.07	5.20	5.58	0.0973
	SD	0.49	1.15	1.25	0.86	1.15	1.39	0.94	1.31	0.55	0.71	
Protein (%)	Mean	3.12	2.83	3.00	3.19	3.38	3.36	3.43	3.41	3.32	3.41	<0.001
	SD	0.28	0.18	0.25	0.24	0.23	0.40	0.27	0.21	0.20	0.27	
Casein (%)	Mean	2.62	2.39	2.57	2.72	2.86	2.88	2.90	2.89	2.80	2.88	<0.001
	SD	0.20	0.14	0.22	0.20	0.17	0.29	0.23	0.16	0.16	0.21	
C14:0 (g/100 g)	Mean	0.44	0.48	0.58	0.63	0.63	0.68	0.55	0.51	0.52	0.57	<0.05
	SD	0.06	0.10	0.14	0.10	0.14	0.15	0.11	0.14	0.06	0.08	
C16:0 (g/100 g)	Mean	1.32	1.40	1.60	1.69	1.64	1.80	1.45	1.36	1.36	1.49	0.0817
	SD	0.20	0.31	0.38	0.29	0.40	0.48	0.32	0.42	0.17	0.25	
C18:0 (g/100 g)	Mean	0.57	0.49	0.50	0.45	0.43	0.44	0.36	0.37	0.37	0.41	<0.001
	SD	0.07	0.11	0.07	0.05	0.06	0.11	0.05	0.09	0.04	0.05	
C18:1 (g/100 g)	Mean	1.51	1.35	1.53	1.49	1.42	1.46	1.31	1.29	1.38	1.41	0.599
	SD	0.17	0.27	0.36	0.19	0.20	0.27	0.19	0.32	0.14	0.13	
LCFA (g/100 g)	Mean	1.91	1.72	2.00	1.92	1.82	1.87	1.64	1.61	1.76	1.80	0.3354
	SD	0.21	0.39	0.39	0.23	0.26	0.35	0.23	0.42	0.17	0.17	
MCFA (g/100 g)	Mean	1.94	2.02	2.32	2.49	2.47	2.71	2.23	2.09	2.07	2.24	0.0292
	SD	0.24	0.41	0.53	0.42	0.56	0.61	0.46	0.54	0.24	0.34	
MUFA (g/100 g)	Mean	1.38	1.22	1.44	1.41	1.34	1.39	1.21	1.21	1.32	1.38	0.4803
	SD	0.17	0.27	0.36	0.19	0.20	0.29	0.18	0.33	0.13	0.14	
PUFA (g/100 g)	Mean	0.15	0.13	0.15	0.13	0.13	0.13	0.12	0.12	0.13	0.14	0.0858
	SD	0.02	0.03	0.03	0.02	0.01	0.02	0.02	0.03	0.01	0.02	
SFA (g/100 g)	Mean	3.60	3.59	4.08	4.26	4.16	4.44	3.65	3.40	3.44	3.71	0.0816
	SD	0.44	0.77	0.85	0.64	0.88	1.04	0.73	0.92	0.40	0.52	
SCFA (g/100 g)	Mean	0.76	0.74	0.82	0.85	0.83	0.87	0.72	0.67	0.66	0.72	0.0665
	SD	0.08	0.16	0.17	0.13	0.17	0.20	0.15	0.18	0.09	0.10	
TFA (g/100 g)	Mean	0.10	0.06	0.11	0.08	0.07	0.06	0.05	0.07	0.06	0.09	<0.05
	SD	0.03	0.03	0.05	0.03	0.02	0.03	0.02	0.05	0.01	0.03	

SD, standard deviation; LCFA, long-chain fatty acid; MCFA, medium-chain fatty acids; MUFA, monounsaturated fatty acids; PUFA, polyunsaturated fatty acids; SFA, saturated fatty acids; SCFA, short-chain fatty acids; TFA, trans fatty acids.

**Table 4:** Correlations of miR-106b, miR-191, and miR-30d relative expression levels with milk constituents of the Holstein and Ayrshire breeds

Constituents	Holstein breed			Ayrshire breed		
	
	miR-106b	miR-191	miR-30d	miR-106b	miR-191	miR-30d
Fat (%)	−0.205	−0.121	−0.049	0.033	0.064	0.031
Protein (%)	−0.062	0.097	0.132	0.343^***^	0.137	0.411^***^
Casein (%)	−0.058	0.114	0.147	0.351^***^	0.154	0.424^***^
C14:0 (g/100 g)	−0.1	0.055	0.105	0.159	0.15	0.13
C16:0 (g/100 g)	−0.173	−0.062	−0.015	0.032	0.096	0.019
C18:0 (g/100 g)	−0.201	−0.270^**^	−0.289^**^	−0.321^**^	−0.136	−0.250^*^
C18:1 (g/100 g)	−0.219^*^	−0.243^*^	−0.198	−0.023	−0.043	−0.003
LCFA (g/100 g)	−0.204	−0.219^*^	−0.193	−0.032	−0.044	−0.019
MCFA (g/100 g)	−0.192	−0.061	−0.002	0.084	0.114	0.085
MUFA (g/100 g)	−0.165	−0.18	−0.141	0.036	−0.051	0.026
PUFA (g/100 g)	−0.036	0.031	0.059	−0.12	−0.143	−0.064
SFA (г/100 г)	−0.225 ^*^	−0.119	−0.049	0.008	0.076	0.027
SCFA (г/100 г)	−0.248 ^*^	−0.14	−0.065	−0.075	0.024	−0.011
TFA (г/100 г)	0.115	0.128	0.084	−0.09	−0.301^**^	−0.127

LCFA, long-chain fatty acid; MCFA, medium-chain fatty acids; MUFA, monounsaturated fatty acids; PUFA, polyunsaturated fatty acids; SFA, saturated fatty acids; SCFA, short-chain fatty acids; TFA, trans fatty acids.

* p<0.05,

** p<0.01,

*** p<0.001.

**Table 5:** MiRNAs and their reactome pathways and predicted target-genes

miRNAs	Total number of reactome pathways	Reactome pathways, associated with milk composition	microRNA target gene	Associated genes
miR-106b	97	Metabolism of lipids BTA-556833 25,360,233,5271	*GPAM*	*MVK, DEGS1, ACSL6, GBA, MGCL, SELENOI, PON3, BTPS2, ORMDL3, LPGAT1, SRD5A1, SACM1L, MTMR2, ACER3, RAB14, ASB12, PCYT1B, PLEKHA8, ACSL3, MORC2, PHYH, PIK3CA, CSNK2A1, M6PR*
		R-BTA-392499 Metabolism of proteins 50,1085,233,5271	*ST8SIA3*	*NOD2, FSTL1, RAD52, CCT6A, STX17, CNIH1, NAPB, RAD23B, CHCHD1, APC, RAB30, RAB31, FBXO21, UBE2E3, RAB33B, TECTB, ST3GAL1, OGT, ST8SIA2, NUP93, SRP14, MRPL51, FEM1C, FBXO11, KCTD7, INO80C, RAB8A, GNG7, RAB14, ABRAXAS2, NUP210, ASB12, MRPL10, MYSM1, ING2, CHM, TRAF3, RAB5B, NR5A2, USP37, DPP4, FSHB, OPCML, SEC11A, CSNK2A1, SPON1, DERL1, GCNT3, FGF23*
		R-BTA-8978868 Fatty acid metabolism 5,94,233,5271	*ACSL6*	*PON3, ACSL3, MORC2, PHYH*
		R-BTA-71291 Metabolism of amino acids and derivatives 7,155,233,5271	*PYCR1*	*SLC25A21, TDO2, AASS, DBT, SERINC5, SLC25A10*
miR-191	41	R-BTA-556833 Metabolism of lipids 6,360,140,5271	*PLPP6*	*SLC22A5, ASAH1, CPNE3, PCYT1A, COL4A3BP*
		R-BTA-392499 Metabolism of proteins 15,1085,140,5271	*EXOC4*	*BABAM2, MRPL34, TUBA1A, USP22, RPL21, PHC3, COPZ1, IKBKG, USO1, CKAP4, SPTB, LMAN1L, THRB*
miR-30d	61	R-BTA-556833 Metabolism of lipids 11,360,136,5271	*CYP51A1*	*FITM2, TNFAIP8L1, LOC782061, ELOVL3, PRKAB2, GPAT3, MBOAT1, PIK3R1, ARSI, SAMD8*
		R-BTA-392499_ Metabolism of proteins 31,1085,136,5271	*ESR1*	*PSMD13, RPL35, TMED9, SHISA5, FEM1A, NAPB, KDELR2, RNF20, USP12, MRPS2, RCE1, DDX17, PRMT3, PTP4A2, RANGAP1, DPH3, GNE, HLTF, BARD1, USP28, ACTR1A, TRAF3, NUS1, GCG, SEC11A, GNB1, FGF23, ARSI, WSB1, PSME3*
